# Particulate Filler and Discontinuous Fiber Filler Resin Composite’s Adaptation and Bonding to Intra-Radicular Dentin

**DOI:** 10.3390/polym15153180

**Published:** 2023-07-26

**Authors:** Marco Ferrari, Eugenia Lettieri, Denise Irene Karin Pontoriero, Pekka Vallittu, Edoardo Ferrari Cagidiaco

**Affiliations:** 1 Department of Prosthodontics and Biomaterials, University of Siena, 53100 Siena, Italy; 2 Department of Oral Surgery, University of Siena, 53100 Siena, Italy; 3 Department of Biomaterials Science, University of Turku, Wellbeing Services County of South-West Finland Turku, 20014 Turku, Finland

**Keywords:** fiber-reinforced composite post, short-glass fiber reinforced composite, endodontically treated teeth, intra-radicular adhesion, push-out bond strength

## Abstract

The aim of this study was to assess adaptation and bonding to root canal dentin of discontinuous (short) glass fiber-reinforced composite to intra-radicular dentin (DSGFRC). Methods: Seventy virgin human teeth were extracted and then endodontically treated; then samples were randomly divided into 7 groups (n = 10), based on the materials’ combinations as follows: Group 1, a two-bottle universal adhesive + DSGFRC; Group 2, a single-component universal adhesive + DSGFRC; Groups 3 and 4, the same materials of Goups 1 and 2 were used but after cleaning of the canal walls with 17% EDTA and final irrigation with 5.25% NaOCl Ultrasound Activated (UA); Group 5, traditional prefabricated fiber posts were luted after being silanized with G-Multi Primer; Groups 6 and 7, like Group 5 but after ultrasonic irrigation (UA). All sample roots were cut 1 mm thick (n = 10) to be evaluated regarding root canal adaptation using a light microscope and scanning electron microscope (SEM) and push-out bond strength. These results were statistically analyzed by Kruskal–Wallis analysis of variance by ranks. The level of significance was set at *p* < 0.05. Results: Bond strength forces varied between 6.66 and 8.37 MPa and no statistically significant differences were recorded among the groups. By microscopic examination, it was noted that ultrasonic irrigation increased the adaptation of the materials to the dentin surface. Conclusions: Within the limitations of this in vitro study, it may be concluded that when DSGFRC was used for intracanal anchorage in the post-endodontic reconstruction, similar push-out retentive force and strength to those of traditional fiber posts cemented with particulate filler resin composite cements were achieved.

## 1. Introduction

The seal of the restoration made on top of endodontically treated teeth is the key for a long positive prognosis [1]. The endodontic treatment makes the tooth weaker from a biomechanical point of view when compared with vital teeth [2]. This structural fragility is due to pathology, access cavity preparation, and excessive removal of dentine during root canal treatment, rather than reduced tissues moisture [3] and absence of cross-linking between dentinal collagen [4]. The loss of strategic components (i.e., ridges, supporting dentin) that can hold occlusal forces preventing tooth fractures is the main reason why ET teeth are vulnerable and show reduced resistance to fracture [5,6]. Wu et al. observed an increase in cuspal deflection as a result of the removal of both marginal ridges in a Mesial–Occlusal–Distal (MOD) cavity preparation and in conjunction with an endodontic access cavity [7]. This is especially important in the case of ET maxillary premolars: these elements are exposed to a combination of shearing and compressive forces, which makes them especially prone to fracture [8]. Therefore, an adequate restorative approach must fulfill both esthetics and the structural preservation and reinforcement of these teeth, as well as providing a coronal seal to protect the endodontic system from bacterial infiltration of the oral cavity. Fiber-reinforced composite (FRC) posts have been widely investigated and used for the restoration of endodontically treated teeth with a significant loss of coronal tooth structure [9,10]. To provide optimal retention and enhanced stress distribution within the root, particulate filler resin composite cements are used as luting agents [11]. The similarity between the modulus of elasticity of the fiber-reinforced composite (FRC) and of root dentin is the solid background that made the use of FRC to restore ET teeth popular among practitioners [12,13]. There are several articles that report the increase of fracture resistance of ET premolars [14,15]. However, the high shrinkage stress related to the unfavorable C factor inside the root canal and the limited adhesion to root canal dentin limit the predictability of luting a post into a root canal [16,17]. Consequently, the most frequent failure is debonding of the post [17].

DSGFRC has been introduced with the claims of better mechanical properties, especially fracture toughness and curing stress behavior, than traditional composites [18]. These materials provided post retention values comparable to traditional dual-cure resin composite cements [19,20]. The high mechanical properties of DSGFRC were shown when used in extremely damaged anterior ET teeth [21] in ET premolar teeth [22]. For that, there is the need to have more information about the use of DSGFRC when used to restore ET teeth. Thus, the aim of the present investigation is to assess, with the push-out test and microscopic examination, the bonding and adaptation characteristics to intra-radicular dentin when DSGFRC was used [23]. For the DSGFRC, improved bonding and toughness properties have been reported, due to high aspect ratio glass fiber fillers and the formation of a semi-interpenetrating polymer network taking place during polymerization [18,24]. The tested null hypothesis was that DSGFRC can have a similar bond to root canal as luted fiber posts.

## 2. Materials and Methods

### 2.1. Specimen Preparation

Seventy single-rooted human teeth, extracted for periodontal or orthodontic reasons, were selected for the study. The freshly extracted teeth were disinfected in hydrogen peroxide solution for 5 min. The soft tissue covering the root surface was removed with hand scalers (Hu Friedy, Chicago, IL, USA). The inclusion criteria (virgin teeth were required) were the visual and radiologic absence of caries or root cracks, previous endodontic treatments, posts or crowns, or resorptions. Teeth were numbered, radiographed, and stored in physiological solution. The exclusion criteria were based on teeth with restorations, decay, or fractures.

The access cavity preparation was carried out with a round-end diamond bur (No. #12; Coltene Whaledent, Altstätten, Switzerland) with water cooling and root canal treatment was performed. The root canal entries were found through an endodontic probe DG16 (Hu Friedy, Chicago, IL, USA); the working length was established by introducing a number 10 K-file (Maillefer-Dentsply, Ballaigues, Switzerland) until it was visible through the apical foramen.

The canals were instrumented with a simultaneous preparation technique by the same operator using nickel–titanium rotary instruments Mtwo (Sweden & Martina, Cornegliana, Italy) sequenced in order (10.04, 15.05, 20.05, 25.06), at the working length. The instruments were used at a speed of 250 rpm, mounted on an endodontic motor (X-SMARTTM Plus; Dentsply Maillefer, Ballaigues, Switzerland), selecting the ProTaper function in the settings. During preparation, the canals were irrigated after every instrument with 5.25% NaOCl solution. Finally, manual gauging was performed with a K-file of the same size as the last rotary instrument that worked at the apex and 5 mm of ethylenediaminetetraacetic acid at 17% (OGNA LAB S.r.l., Muggiò, Italy) was left for a total time of two minutes; the final irrigation was then carried out with 5 mm of NaOCl at 5.25%.

The preformed cone of gutta-percha (Mtwo, Sweden & Martina, Le Duecarrare, Italy) of the same diameter and taper of the last rotary instrument that worked in the apex was tested, checking for slight resistance (tug back). The canals were dried with paper cones (Dentsply Sirona, Charlotte, NC, USA).

Obturation was performed using a continuous wave condensation technique with gutta-percha cones (Mtwo, Sweden & Martina) cut at the apex (0.5 mm) and a root canal cementation material based on zinc oxide/eugenol (Pulp Canal Sealer, Kerr, Berlin, Germany). The cone was condensed and compacted 5 mm from the apex (down packing). Roots were then back-filled with thermoplastic injectable gutta-percha (Obtura, Meta systems EQ-V, Algonquin, IL, USA), then compacted with an endodontic plugger (Machtou 1–2. Dentsply Sirona, Charlotte, NC, USA). Postoperative periapical radiographs were taken for all samples.

All root canal-treated teeth received a post space preparation: part of this filling material was removed with low-speed Number 5 and 6 Gates Glidden burs; the canal walls were enlarged with low-speed Number 5 and 6 Largo (Dentsply Maillefer, Ballaigues, Switzerland) burs, leaving a minimum apical seal of 4–6 mm of gutta-percha in the canal. The postoperative X-rays were performed for the second time. The depth of the root canal space to be filled was up to 4–5 mm to the anatomic apex. The chemical composition of the materials used in this study is reported in Table 1.

Samples were randomly divided into 7 groups, as follows:

1. G2 Bond Universal (GC Corporation, Tokyo, Japan) + EverX Flow (GC Co.)
2. G-Premio Bond (GC Co.) + EverX Flow (GC Co.)
3. G2 Bond Universal (GC Co.) + EverX Flow (GC Co.) with ultrasonic activation (UA).
4. G-Premio Bond (GC Co.) + EverX Flow (GC Corporation) with ultrasonic activation (UA).
5. G-Premio Bond (GC Co.) + a dual-cured composite resin cement (GradiaCore, GC Co.) + GC Fiber Post 1.6 mm (GC Co.), silanized with G-Multi Primer for 1 min (GC Co.)
6. G-Premio Bond (GC Co.) + a dual-cured composite resin cement (GradiaCore, GC Co.) + GC Fiber Post 1.6 mm (GC Co.), silanized with G-Multi Primer for 1 min (GC Co.) with ultrasonic activation (UA).
7. G2 Bond Universal + prefabricated FRC post (GC Co.) with ultrasonic activation (UA).

In groups 1, 2, and 5, root dentin was cleaned with 17% EDTA for 30 s and then a final irrigation with 5.25% NaOCl. The same protocol was performed in groups 3, 4, 6, and 7 but using ultrasonic activation of irrigant solutions (NEWTRON P5 XS; Satelec Acteon, St. Egeve, France). The root canals were dried with paper points. The teeth received different adhesive treatments:

- In groups 1, 3, and 7, a two-bottle universal adhesive (primer + bonding) G2 Bond Universal (GC Co.) was used according to the manufacturer’s instructions. The primer solution was applied in the canal with a microbrush (GC Co.). Excess adhesive was removed from the post space with gentle air blowing and absorbent paper points. After the bonding application, excess adhesive was removed by suction drying and light-cured for 20 s using a LED light (VALO Cordless-LED Curing Light—Ultradent, Milano, Italy).
- In the other groups (2, 4, 5, 6), a one-bottle G-Premio Bond (GC Co.) universal adhesive was applied in a single step, the excess was removed with a gentle air blowing and the adhesive was cured.

After light curing the adhesive, the teeth in groups 5–7 received a conventional translucent glass FRC post (GC Fiber Posts, GC Co.) of 1.6 mm diameter. The posts received silanization of the surface (G-Multi Primer, GC Corporation) for 1 min following the manufacturer’s recommendation. Luting of the posts was performed with a dual-cured composite resin cement (Gradia Core, GC Co.). Gradia Core was applied using its own automix cartridge. The post was seated with a slight finger pressure. The cement excess was removed by cotton pellets and light-cured through the post for 20 s with an LED, keeping the light tip in contact with the end of the post.

In the other groups (1, 2, 3, 4), no fiber posts were used, whereas after the application the adhesive system, the DSGFRC was injected into the post space with the use of DSGFRC compule and light-cured for 20 s, keeping the light tip in contact with the coronal opening of the cavity.

In order to reduce possible bias, all the restorative procedures were performed by the same operator.

### 2.2. Push-Out Loading Test

Crowns were removed at the cement–enamel junction (CEJ), using a water-cooled diamond disc (Isomet, Buehler, New York, NY, USA). All the roots were sectioned perpendicularly to their long axis by means of an Isomet saw under water cooling (Buehler), providing 1 mm thick slices for each sample and the slides were made starting 2 mm below the CEJ.

**Figure 1:**
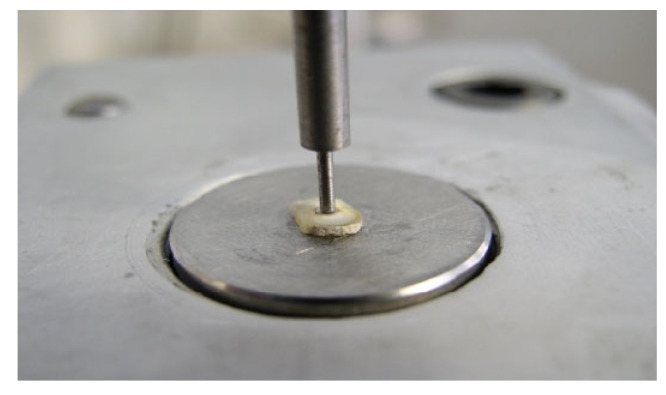
Push-out force applied to the apical aspect of the slice via a cylindrical plunger mounted on a universal testing machine.

To evaluate the bonding properties, the push-out test was performed using a universal testing machine (Triax 50, Controls SPA, Milano, Italy), at a cross-head speed of 0.5 mm min until bond failure occurred, as manifested by the extrusion of the post/composite segment from the root slice. On the loading machine, each slice was positioned with the apical side of the segment facing the plunger tip, so as to apply the loading force in the apical–coronal direction. The plunger tip was sized and positioned to touch only the post or the composite, without stressing the surrounding root canal walls (Figure 1). Testing was carried out for water-conditioned samples at room temperature.

A digital caliper with 0.01 mm accuracy was used to measure the thickness of each slice, as well as the coronal and apical diameters of the posts/composites. The retentive force was measured and corresponding bond strength of the post segment was expressed in MPa, by dividing the load at failure in Newtons and the bonded interfacial area (A, mm^2^) of the post fragment. The interfacial area was calculated as the lateral surface of a truncated cone using the formula A = π(R + r)[h^2^ + (R − r)^2^]^0.5^, where π = 3.14, R is the coronal post radius, r is the apical post radius, and h is the thickness of the slice.

### 2.3. Microscopic Analysis

In addition, to observe the type of failure at the adhesive interfaces and post-curing adaptation of adhesive resin or luting cement in the different groups, the specimens were firstly visually examined under a stereomicroscope (Nikon H550L, Nikon, Tokyo, Japan) and then subjected to scanning electron microscopy (SEM) observation. Prior to SEM observation, all the specimens were gold-coated using a sputter coater in a vacuum evaporator (Emitech K550 Sputter Coater, New York, NY, USA). For the SEM evaluation, a low vacuum scanning electron microscope (Joel, Joel Co., Tokyo, Japan) with a voltage of 15 kV, using secondary electrons (SE), was used. Failures were classified as follows: A: Adhesive failure at the dentin–cement interface; C: Cohesive failure within the restorative material; M: Mixed failure at the dentin–cement interface and cement–restorative material interface.

### 2.4. Statistical analysis

As the data distribution was not normal, the Kruskal–Wallis analysis of variance by ranks had to be applied. The level of significance was set at *p* < 0.05 and calculations were performed using the SigmaPlot software for Windows (version 11.0).

The expected percentage of successful treatments, arbitrarily set at 98%, was used as the basis to calculate the needed sample size to identify group difference with the conventional 5% type I error and 20% type II error. Based on that, 33 samples per group were needed to have an 80% statistical power.

## 3. Results

Results of push-out force (N) and strength (MPa) to debond the material from the root canal dentine are reported in Table 2. Type of failure is reported in Table 3.

When UA was applied before the bonding step, some Cohesive failures (C) were noted, whilst in the other groups, only Adhesive (A) and Mixed (M) failures were recorded.

Kruskal–Wallis tests revealed no statistically significant differences among the groups (*p* = 0.902). In general, group 3 recorded the highest values of bond strength, while group 2 recorded the lowest values.

**Figure 2:**
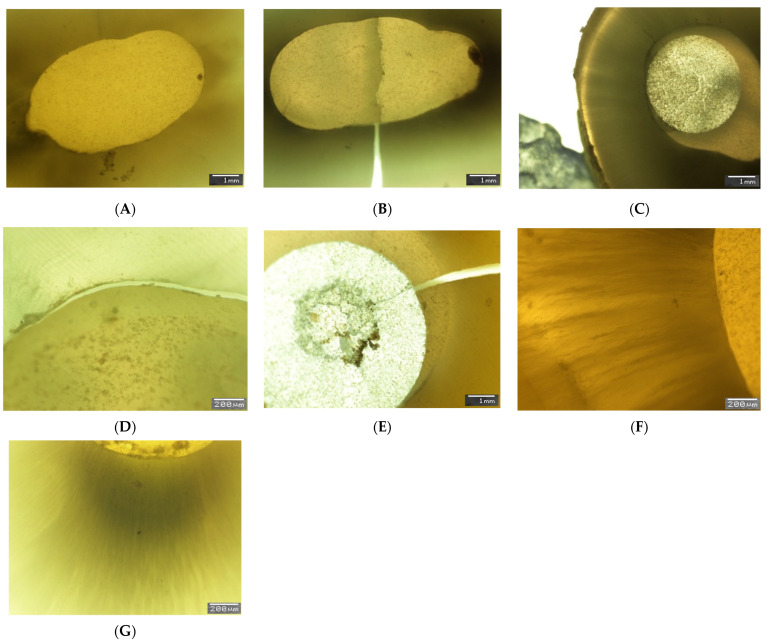
(**A**). Group 1: DSGFRC filled the root canal properly. (**B**). Group 2: Cohesive failure of DSGFRC under loading. (**C**). Group 3: Thick thickness of cement between the post and the radicular dentin. (**D**). Group 4: Adhesive failure at the interface between radicular dentin and DSGFRC. (**E**). Group 5: Cohesive failure of a fiber post. (**F**). Group 6: The material adapted well to the dentin surface (G-Premio Bond adhesive system after ultrasonic activation). (**G**). Group 7: The resin material penetrating deeply into the dentinal tubules (G2 Bond adhesive system after ultrasonic activation).

**Figure 3:**
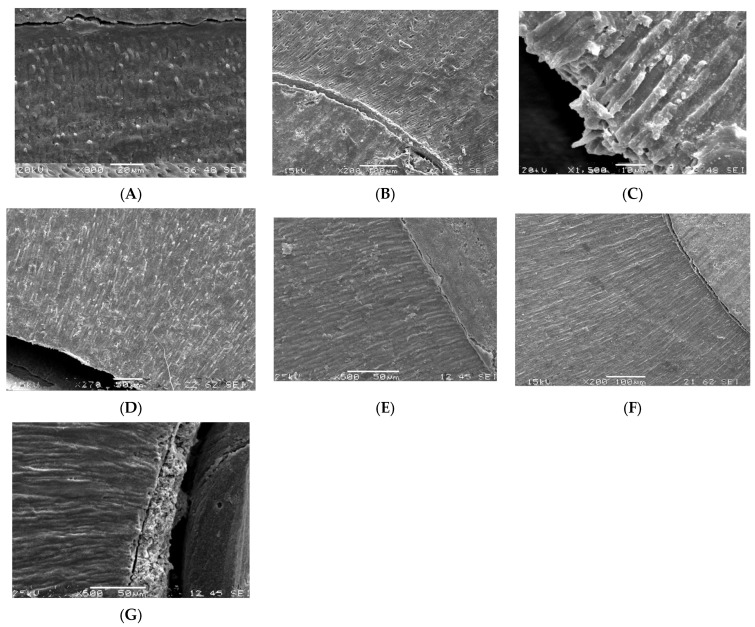
(**A**). Group 1: short resin tags (G2 Bond) are visible and adhesive failure type (SEM x800). (**B**). Group 2: no resin tags (G-Premio Bond) are visible and adhesive failure type (SEM x200). (**C**). Group 3: Resin tags (G2 Bond) formation after ultrasonic activation (SEM x1200). (**D**). Group 4: Resin tags (G-Premio Bond) after ultrasonic activation and adhesive failure (SEM x270). (**E**). Group 5: Mixed failure (SEM x500). (**F**). Group 6: Resin tags formation (G-Premio Bond) after ultrasonic activation and adhesive failure (SEM x300). (**G**). Group 7: Mixed failure sample; the SEM picture shows the debonding at cement and post interface (SEM x500).

The shots taken at the SEM are shown in Figure 2A–G and Figure 3A–G. Cohesive failures were noted only when ultrasonic activation was used (Figure 2B,E). In all other groups, the type of failure was distributed between adhesive (Figure 2D) and mixed (Figure 2C).

The microscopic analysis showed improved post-curing adaptation of luting materials in the ultrasonically activated groups (Figure 2F,G and Figure 3A–D,F).

## 4. Discussion

In this study, DSGFRC showed statistically equal intra-radicular bond strength to radicular dentin to those of traditional prefabricated fiber post groups. This led to the acceptance of the null hypothesis.

Fiber posts have a modulus of elasticity pretty similar to that of the root dentin, and for that, the risk of root fracture is considerably low [25,26], along with high aesthetic outcomes of the restorative treatment [27]. However, due to complexity of adhesive-luting steps, it must be considered that luting procedures are technique-sensitive and related to the operator skill and knowledge [28]. Moreover, the placement of FRC posts often implies extensive removal of the root dentin, which is a major drawback since tissue preservation is strongly associated with the survival of endodontically treated teeth [29,30]. The resin composite core-built-up material and luting cement in combination with a prefabricated glass fiber post shows comparable bonding properties but higher material cohesive strength properties than actual resin composite luting cements on the market. In fact, even the four-year follow-up work [31] demonstrated the advantageous properties of the core material.

Despite the properties and bonding techniques of more modern resin-based materials, the most common type of failure when using fiber posts remains the debonding of the post at the post-dentin interface [32]. Some clinical aspects can determine the debonding [33], such as silanization of post surface, bonding steps and its polymerization, the possibility of an incomplete polymerization of bonding luting materials into the deepest part of the root canals, and the shape of the root canal [26,34,35]. When the post does not seat completely, a tick thickness of luting materials will be into the most apical part of the root canal and voids and bubbles might be present, predisposing to debonding. Moreover, the transmission of the light through the fiber post is another factor that can determine debonding of the post [36,37,38]. When a fiber post is luted, the bond strength to root dentin is low because of the high cross-link density of the polymer matrix of the post [37].

The use of DSGFRC, a discontinuous glass fiber-reinforced resin composite, as intra-radicular filling and post replacing material permits to eliminate some of the variables which may increase technique sensitivities of prefabricated fiber posts. The use of injectable composite with short fibers and related increased fracture toughness of the material and simplified handling and clinical steps may increase clinical success of the treatment. Attention should be paid to the post-curing adaptation of the luting and other resin-based materials to the dentin surface. The translucent shade of DSGFRC polymerizes to the depth of 5 mm and the discs of measuring the bonding properties and post-curing adaptation of the composite in the present study were cut in the area of polymerized composite. A root canal of depth of 5 mm with thick root canal post has shown to provide better load-bearing capacity for the restoration than thinner prefabricated posts with higher post length [39,40].

The use of DSGFRC does not need any post-space preparation [41,42]. Therefore, adaptation to the root canal anatomy, without additional dentin removal, may be of advantage for tissue preservation.

The push-out test is a critical test to measure the axial bond strength of a post-retained restoration bonded to root canal dentin [11]. In this study, DSGFRC tested for intracanal anchorage achieved statistically similar push-out bond strength values to those of a traditional fiber post system with particulate filler resin composite. This recent material consists of a combination of a resin matrix, discontinuous E glass fibers, and inorganic particulate fillers. The resin matrix contains cross-linked monomers, bisphenol-A-glycidyl dimethacrylate (bis-GMA), and triethylene glycol dimethacrylate (TEGDMA). The composition differs from the composition of continuous unidirectional fiber-reinforced composite, which is used in individually formed fiber posts which also contain linear polymethyl methacrylate (PMMA) and form a semi-interpenetrating polymer network (semi-IPN) on curing, which is demonstrated to improve bonding and toughness properties of the composite resin [18]. The formation of semi-IPN bonding is not needed with discontinuous fiber composite of DSGFRC in the root canal application because free radical polymerization of the composite allows simultaneously good bonding of the adhesives.

The reinforcing effect of the fiber fillers having high enough aspect ratio (typically >20) and critical fiber length which relates to the fiber adhesion to the polymer matrix is based on stress transfer from polymer matrix to fibers and individual fibers as a crack stopper to hinder crack growth. Discontinuous and randomly oriented fibers provide an isotropic reinforcing effect; specifically, the strength of the material is independent of the fracture load direction and is comparable in all directions [18,41,42]. Moreover, an inherently uniform stress distribution to the hard biological tissues has also been documented for DSGFRC, mainly due to fracture toughness values matching those reported for dentin [43]. It is also likely that the discontinuous glass fibers of a diameter of 5–6 μm could provide micromechanical interlocking to the dentine surface irregularities which could increase adhesive properties in shear stress situations.

This study also evaluated the bond strength to intra-radicular dentin of a two-step self-etch adhesive (G2 Bond Universal) compared to a universal adhesive (G-Premio Bond) showing no statistical significant differences.

The application of a two-step self-etch adhesive system that provides the use of an additional layer of hydrophobic resin achieved a stronger bond strength to root dentin than one-step self-adhesive bonding systems [44,45].

The one-step universal adhesive G-Premio Bond contains highly volatile acetone which evaporates quickly, leaving water behind. Too much remaining water can contribute to incomplete polymerization, culminating in a weak interface and premature bond failure [46,47]. G2-Bond Universal adhesive is a two-bottle system but has a HEMA-free composition similar to G-Premio Bond [48]. Thanks to its two-bottle strategy and UDMA in the bonding resin, G2-Bond Universal provides a more hydrophobic bond layer [48,49] which imparts a better shock-absorbing effect against shrinkage stress [50]. The use of the two-step adhesive system also leads to a lower number of adhesive failures at the bonding interfaces.

In addition to external factors such as irrigants, types of adhesives, endodontic sealers and factors related to dentin, intracanal adhesion depends on the removal of the smear layer and the creation of a hybrid layer between the root canal and the adhesive resin [51]. The smear layer can be removed using a combination of chelating agents and NaOCl [52,53]. As a complement to various irrigant solutions, ultrasound contributes to the elimination of the smear layer [54,55]. The microscopic analysis showed more even adaptation and possibly even extended resin tags on the root surface in the ultrasonic irrigant activated groups. Moreover, the push-out test revealed that irrigant activation affects the bond strength to intra-radicular dentin.

The combination of UA of EDTA and NaOCl, G2 Bond Universal, and DSGFRC showed a similar bond strength to intra-radicular dentin when fiber posts were used, but a deep penetration of resin tags into the tubules and more favorable type of failure. In addition, when UA was applied before the bonding step, some Cohesive failures were noted, whilst in the other groups, only Adhesive and Mixed failures were recorded.

## 5. Conclusions

Within the limitations of this in vitro study, it can be concluded that DSGFRC used to restore ET teeth can achieve a similar push-out strength to that obtained by traditional fiber posts. DSGFRC in combination with a two-step adhesive system and ultrasonic irrigation represents a viable and operatively simpler alternative to traditional fiber post adhesion.

## Figures and Tables

**Table 1:** Chemical composition of the materials used in the study.

Material	Composition
G-Premio Bond (GC Co., Tokyo, Japan)	10-MDP, 4-META, 10-MDTP, methacrylate acid ester, distilled water, acetone, photoinitiators, fine powdered silica
G2 Bond Universal (GC. Co.)	Primer: 4-META, MDP, dimethacrylate, photoinitiator, water, acetone, silica, MDTP Bond: Dimethacrylate, photoinitiator, silica
EverX Flow (SFRC) (GC. Co.)	Bis-EMA, TEGDMA, UDMA, micrometer scale glass fiber filler 100–300 μm and Ø7 μm, Barium glass 70 wt%, 46 vol%
Gradia Core (GC. Co.)	Methacrylic acid ester 20–30 wt%, fluoro-alumino-silicate glass 70–75 wt%, silicon dioxide 1–5 wt%.
GC Fiber Post (GC. Co.)	Glass fibers, dimethacrylate matrix
G Multi Primer (GC. Co.)	MPTMS, 10-MDP, MDTP, BisGMA, TEGDMA, Ethanol

10-MDP, 10-methacryloyloxydecyl dihydrogen phosphate; 4-META, 4-methacryloxyethyl trimellitic anhydride; 10-MDTP, 10-methacryloxydecyl dihydrogen thiophosphate; UDMA, urethane dimethacrylate; TEGDMA, triethyleneglycol dimethacrylate; Bis-EMA, Ethoxylated bisphenol-A- dimethacrylate; Bis-MEPP, 2,2-bis(4 methacryloxypolyethoxyphenyl) propane; PMMA, polymethyl methacrylate; wt%, weight percentage; MPTMS, methacryloxypropyl trimethoxysilane; BisGMA, bisphenol-A glycidyl dimethacrylate.

**Table 2:** Descriptive statistics of push-out force (in N) in the groups; Kruskal–Wallis tests revealed no statistically significant differences among the groups (*p* = 0.902).

Group	N	Median	Interquartile Range
1	44	7.35	5.35–11.11
2	38	6.66	5.87–8.75
3	36	8.37	6.46–10.55
4	43	7.72	5.34–10.71
5	33	7.14	4.65–10.31
6	42	6.78	4.49–13.59
7	39	7.99	4.62–8.92

**Table 3:** Type of failure: N: number of samples; A: Adhesive failure at the dentin–cement interface; C: Cohesive failure within the restorative material; M: Mixed failure at the dentin–cement interface and cement–restorative material interface.

Group	N	A	C	M
1	44	20	/	24
2	38	23	/	15
3	36	10	7	19
4	43	18	2	23
5	33	14	/	19
6	42	12	1	29
7	39	22	4	13

Group 1: G2 Bond Universal + EverX Flow. Group 2: G-Premio Bond + EverX Flow. Group 3: ultrasonic activation (UA) of EDTA and NaOCl (UA)+ G2 Bond Universal + EverX Flow. Group 4: UA + G-Premio Bond + EverX Flow. Group 5: G-Premio Bond + prefabricated FRC post. Group 6: UA + G-Premio Bond + prefabricated FRC post. Group 7: UA + G2 Bond Universal + prefabricated FRC post.

## Data Availability

Not applicable.
